# Intratumor fungi specific mechanisms to influence cell death pathways and trigger tumor cell apoptosis

**DOI:** 10.1038/s41420-025-02483-z

**Published:** 2025-04-21

**Authors:** Simran S. Ghogare, Ejaj K. Pathan

**Affiliations:** Symbiosis School of Biological Sciences, Symbiosis International (Deemed University) Lavale, Pune, 412115 Maharashtra India

**Keywords:** Antifungal agents, Drug development

## Abstract

Cancer, uncontrolled cell growth due to the loss of cell cycle regulation, is often found to be associated with viral infections and, as recent studies show, with bacterial infections as well. Emerging reports also suggest a strong link between fungi and cancer. The crucial virulence trait of fungi, the switch from yeast (Y) to hyphal (H) form, is found to be associated with carcinogenesis. The physicochemical properties and signal transduction pathways involved in the switch to the hyphal form overlap with those of tumor cell formation. Inhibiting differentiation causes apoptosis in fungi, whereas preventing apoptosis leads to cancer in multicellular organisms. Literature on the fungi-cancer linkage, though limited, is increasing rapidly. This review examines cancer-specific fungal communities, the impact of fungal microbiome on cancer cell progression, similarities between fungal differentiation and cells turning cancerous at biochemical and molecular levels, including the overlaps in signal transduction pathways between fungi and cancer. Based on the available evidence, we suggest that molecules inhibiting the yeast-hyphal transition in fungi can be combined with those targeting tumor cell apoptosis for effective cancer treatment. The review points out fertile research areas where mycologists and cancer researchers can collaborate to unravel common molecular mechanisms. Moreover, antibodies targeting fungal-specific chitin and glucan can be used for the selective neutralization of tumor cells. These new combinations of potential therapies are expected to facilitate the development of target-specific, less harmful and commercially feasible anticancer therapies.

**Figure:**
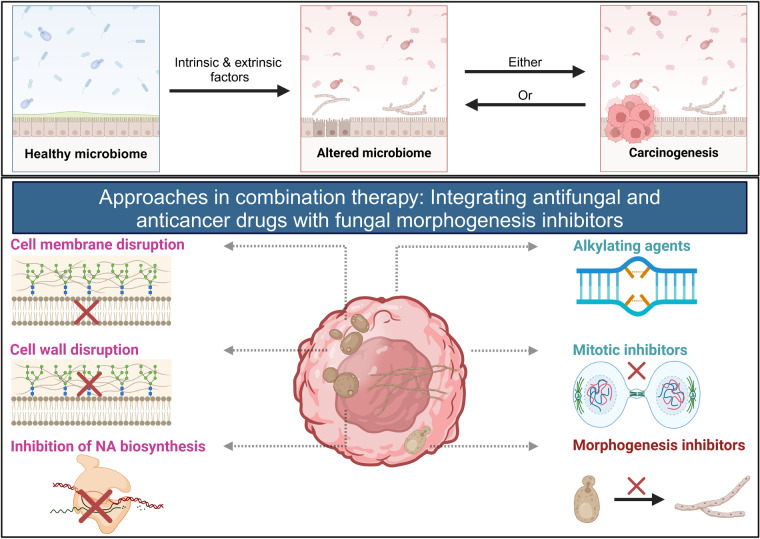
We bring together available evidence to argue that fungal infections could either trigger cancer or have a significant role in the development and progression of cancer. Hence, cancer-associated fungal populations could be utilized as a target for a combination therapy involving the integration of anticancer and antifungal drugs as well as inhibitors of fungal morphogenesis to develop more effective anticancer therapies.

We bring together available evidence to argue that fungal infections could either trigger cancer or have a significant role in the development and progression of cancer. Hence, cancer-associated fungal populations could be utilized as a target for a combination therapy involving the integration of anticancer and antifungal drugs as well as inhibitors of fungal morphogenesis to develop more effective anticancer therapies.

## Facts

- Fungi influence tumor progression through morphology-mediated immune modulation and metabolite production.
- The physicochemical properties and signal transduction pathways involved in fungal morphogenesis overlap with those of tumor cell formation.
- The molecules inhibiting fungal morphogenesis can be combined with existing anticancer agents for effective cancer treatment.
- Antibodies targeting fungal-specific chitin and glucan can be used for the selective neutralization of tumor cells.
- Understanding the crosstalk between tumor-associated fungal communities can help in the prognosis and diagnosis of specific cancer types.

## Open questions

- How do fungal infections contribute to development and progression of cancer?
- Do cancer-type specific fungal signatures overlap with tumor cell progression?
- How do tumor microenvironments facilitate fungal differentiation and immune system evasion?
- Can inhibitors of fungal differentiation induce apoptosis in cancer cells?
- Can dealing with fungi help in development of novel and safe anticancer therapy?

## Introduction

Cancer is among the leading causes of death worldwide, with 20 million new cases and 9.7 million deaths reported globally in 2022 [1]. Discussing the key characteristics of cancer cells, Hanahan et al. list eight hallmarks of cancer, including the ability to sustain proliferative signaling by reprogramming cellular metabolism, thus resisting cell death and enabling replicative immortality. Cancer tissues can evade growth suppressors, avoid immune destruction and induce vasculature for effective food supply. On occasion, they can activate invasion and metastasis. The acquisition of these abilities to trigger neoplastic growth is enabled by genome instability and tumor-promoting inflammation [2].

While various therapeutic approaches, such as chemotherapy, endocrine therapy, thermal ablation, and photodynamic therapy, have proven effective against cancer, they often cause side effects. For instance, chemotherapy is associated with non-selective action against actively proliferating normal cells [3]. Endocrine therapy interferes with normal hormonal secretion; thermal ablation leads to blood clots and tissue damage, and photodynamic treatment is restricted to the treatment of tumors just under the lining of internal organs [4]. Moreover, excess use of anticancer drugs leads to the development of resistance in cancer cells.

Though there may be genetic factors that predispose some people to some cancers, it has been long recognized that viruses may also be involved [5]. The hijacking of the cellular machinery by viruses was proposed to explain the mechanism [6]. Around the same time, the involvement of bacteria in some cancers was reported [7]. Altered bacterial populations have been observed to initiate the synthesis of genotoxins like colibactins (produced by *Escherichia coli*), CDTs (cytolethal distending toxins produced by Gram-negative bacteria), and endonucleases (produced by *Neisseria gonorrhoeae*). These genotoxins were proposed as mechanisms responsible for mediating DNA damage leading to oncogenesis [8]. About a decade later, the involvement of fungi in some cancers was reported [9, 10]. It was assumed that these cases might be due to opportunistic infections and, perhaps, due to toxins, such as aflatoxin, secreted by fungi. However, recent evidence suggests a deeper causative role of fungi in cancers.

The mycobiome, inhabiting barrier surfaces, represents the fungal population of the human microbiota [11]. These fungal species are crucial for maintaining intestinal homeostasis and systemic immunity [12]. Specific host factors can trigger the remodeling of the distribution and composition of these fungal species, causing them to switch from a non-virulent (saprophytic) to a virulent (pathogenic) form, leading to mycobiome dysbiosis [13]. Evidence of the association between mycobiome dysbiosis and cancer has been accumulating in recent years. For instance, fungal spores and hyphae were recovered from the tissue samples of cancer patients. Characterization of the gastric mycobiome in 22 samples of gastric cancer and normal adjacent tissue and 11 non-cancerous normal tissue samples demonstrated that fungi exist in non-virulent morphotypes in normal cells, whereas they switch to virulent morphotypes in tumor microenvironments [14].

The fungi-cancer linkage has evolved from early speculations to concrete scientific evidence. The early discovery of fungi-derived secondary metabolites called aflatoxins (AFs) sparked discussion in the 1960s after an outbreak of turkey “X” disease [15]. Since then, researchers have reported their organotoxic, mutagenic, and carcinogenic effects [16]. AFs such as AFB1, AFB2, AFG1, and AFG2 have been labeled to be carcinogenic to humans by the International Agency for Research on Cancer as they induce the formation of DNA adducts responsible for the increased risk of developing cancer [17]. AFB1, a hepatotoxic compound, is known to induce the expression of autophagy-related protein P62, which has been implicated in developing liver and breast cancer [18, 19]. Later, chronic *Candida* infection and fungal dysbiosis were found to be associated with increased cancer risk, particularly in immunocompromised individuals. Recently, metagenomic analysis revealed fungal DNA in tumors and suggested their role in inflammation-driven carcinogenesis [20]. It indicates that fungi may influence tumor progression through immune modulation and metabolite production.

Moreover, there are a number of physicochemical events that overlap between the process of fungal differentiation and the development and progression of cancer cells [21, 22]. These findings suggest that cancer could either result from fungal infection or that fungal infection has a significant role in the development and progression of cancer. In either case, fungal cells present in the tumor microenvironment can be targeted to trigger programmed cell death or apoptosis of cancer cells. In this context, we review existing literature on the association between fungi and cancer development to provide a comprehensive insight into the possibility of combining antifungals with other therapies for more effective cancer treatment.

## Role of harmless mycobiome in the human body

The human mycobiome, the fungal communities present in and on the human body, varies drastically between two individuals and this diversity originates right at the birth of the infant. The mode of delivery of an infant contributes to early-life mycobiome diversity. Vaginal delivery allows the vertical transmission of *Candida* sp., while cesarean section delivery is characterized by *Malassezia* sp. and lower taxonomic levels [23]. Following delivery, the gut of the infant was found to be dominated by breast milk mycobiome, composed of *Malassezia*, *Davidiella*, *Sistotrema*, and *Penicillium sp*. [24]. Further modifications of mycobiome are associated with growth and developmental stages from infancy to adolescence and are driven by the advancement of the immune system-associated microbial community [25], intrinsic factors [genetics, sex, age], and extrinsic factors [food, medication, hygiene] [26]. These fungal inhabitants play an essential role in maintaining intestinal homeostasis by establishing cross-kingdom interactions. For instance, *Saccharomyces boulardii* has been shown to neutralize *Vibrio cholera* toxins by activating the cyclic AMP pathway in rat models [27]. *Malassezia* and *Candida albicans* prevent infections from opportunistic organisms such as *Aspergillus amstelodami*, *Epicoccoum nigrum* and *Wallemia sebi*. *Aspergillus fumigatus* can synthesize gliotoxin, which prevents biofilm formation by *Pseudomonas aeruginosa* [28]. These fungal species also support the immune system’s ontogenesis by directing the retinol dehydrogenase-positive [RALDH^+^] dendritic cells [DCs] from the intestinal lamina propria to secondary lymphoid organs. These DCs support the development of gut-associated lymphoid tissues [26] and are also responsible for retinoic acid secretion, promoting tolerogenic responses [29].

## Mycobiome dysbiosis in the induction of carcinogenesis

The human mycobiome not only plays a vital role in maintaining human health, but is also implicated in the development of different diseases. Human commensal fungi usually modify pathways related to energy acquisition, metabolic homeostasis, immune and neurological development, etc. and, therefore, are considered crucial for human health [30]. Mycobiome dysbiosis suggests an imbalance in the abundance of these commensal fungi due to the occurrence of pathogenic fungi and may lead to the development and progression of various types of cancer [31]. Studies suggest that mycobiome dysbiosis triggers pro-inflammatory responses, leading to the transformation of epithelial cells and DNA damage, ultimately imparting a carcinogenic effect [32]. One of the mechanisms by which fungi participate in cancer development and progression is by inducing alterations in the immune response. The molecules and strategies used by fungal pathogens to evade the host immune recognition and response are summarized in Supplementary Table S1.

Dectin-1, a key component of the host defense mechanism, is a C-type lectin receptor that can recognize cell wall-associated β-1,3-glucan. Followed by recognition, Dectin-1 promotes the assembly of inflammasomes, activating the production of inflammatory cytokines such as pro-IL-1β and pro-IL-18. The result of this process leads to the activation of NLRP3 inflammasome. This complex triggers the conversion of pro-IL-1β and pro-IL-18 into their active form, IL-1β, and IL-18, respectively [33]. These cytokines are known to regulate the production of IL-22, which ultimately promotes tumor initiation and growth by activating the epithelial signaling transducer and activator of transcription 3 [34, 35].

Another mechanism by which filamentous fungi contribute to cancer progression is through the generation of metabolites like nitrosamines, candidalysin, and acetaldehyde [ACH]. ACH, a mutagenic and carcinogenic factor, activates the Ca^2+^-mediated signaling pathway, phosphorylating dynamic-related protein 1 [Drp1]. As a key regulator of mitochondrial fission, Drp1 translocate to mitochondria and initiates multiple fragmentations responsible for the overproduction of reactive oxygen species [ROS] through heightened respiration and mitochondrial hyperpolarization. This leads to DNA damage, tissue invasion, and metastasis [35, 36]. The aflatoxins, secondary metabolites produced by fungi, also play an important role in the development and progression of cancer [17–19].

The following subsections describe the role of fungal species in different types of cancer development and progression. An attempt is also made to understand the correlation between cancer type and associated fungal species to identify cancer-type-specific fungal communities, if any.

### Fungal species and oral cancer

Benign inhabitant of the oral cavity, *C. albicans* may switch to the pathogenic form [37] in response to heavy alcohol consumption [38, 39]. The fungal ubiquitous enzyme, alcohol dehydrogenase (EC 1.1.1.1), plays an essential role in metabolism and growth [40]. It can metabolize alcohol to a genotoxic carcinogen, ACH [30]. The synthesized ACH forms DNA adducts, obstructing DNA replication [41]. It binds to the antioxidant glutathione, enhancing the production of ROS, and leading to inflammation and mitochondrial damage [42, 43]. It also causes DNA crosslinks, chromosomal aberrations [44], and enhanced Sister Chromatid Exchanges [45]. The accumulation of such genetic alterations could lead to cancer [19].

Another mechanism by which *C. albicans* triggers uncontrolled cell growth is by activating the toll-like receptor [TLR]-MyD88 signaling pathway. Members of the TLR family transmit signals to the tumor promoter, NF-κB, to activate inflammatory cytokines and enzymes such as cyclooxygenase (COX-2, EC 1.14.99.1) [46], which promotes the proliferation, apoptotic resistance, and metastasis of tumor cells [47]. *C. albicans* can also influence the TLR2/MyD88 and TLR2/NF-κB signaling pathways to upregulate the expression of the programmed death-ligand 1 [PD-L1] [48] with the ability to inactivate cancer cell killer T lymphocytes [49]. Researchers have also observed the increased expression of pro-inflammatory cytokines such as IL-6 and IL-8 in the tumor microenvironment [50]. These pro-inflammatory cytokines play a significant role in cancer progression by exerting anti-apoptotic effects on cancer cells. The increased expression of cytokines may be due to fungal cell wall-associated constituents such as phospholipase B, lipase, and aspartyl proteinase secreted by yeast cells [51]. Such hydrolytic enzymes may also contribute to carcinogenesis by enhancing the invasion of host tissues by pathogenic fungal species, first by damaging adhesive E-cadherin between adjacent keratinocytes and then by degrading laminin-332 associated with oral epithelium [46]. *C. albicans* may also colonize existing pre-malignant lesions and promote the progression of cancer by mediating the synthesis of the potent carcinogen, *N*-nitroso benzyl methylamine, from the precursors, *N*- benzyl methylamine and nitrite, at the neutral pH of the oral cavity [52]. It can activate proto-oncogenes and cause dysplasia [19].

### Fungal species and esophageal cancer

In 2020, esophageal cancer led to an estimated 604,100 new cases and 544,076 deaths [1]. Host-associated factors like smoking, alcohol intake, and high starch consumption without adequate fiber can lead to squamous cell carcinoma. In contrast, risk factors for esophageal adenocarcinoma include gastroesophageal reflux disease and obesity [53]. Besides the above-identified risk factors for the two different kinds of esophageal cancers, extensive research has demonstrated the role of fungal species in the development of squamous cell carcinoma in immunodeficient patients suffering from auto-immune polyendocrinopathy candidiasis ectodermal dystrophy [APECED] [54], an autosomal recessive disorder caused by mutations in the autoimmune regulator gene, which encodes the nuclear transcription protein responsible for the induction of immunological self-tolerance [55, 56]. Hence, the defense system of APECED patients fails to eliminate autoreactive T-lymphocytes [57, 58]. A study using kinase dead *Ikkα* knock-in [*Ikkα*^KA/KA^] mice, mimicking similar phenotypic characteristics as seen in APECED patients, demonstrated that autoreactive T-cells permit fungal infection. These alterations, along with elevated levels of the epidermal growth factor receptor [EGFR] and decreased P^53^, P^16^, and Rb- gene expression, are crucial players in developing squamous cell carcinoma [54]. The alterations described above impaired T-lymphocyte-mediated immune response, leading to a defect in the production of immune components such as IL-17, IL-22 (essential for mucosal antifungal defense) and IFN-γ (essential for tumor surveillance). Moreover, they lead to mutations in the signal transducer and activator of the transcription 1 [STAT1] gene that induces esophageal cancer [54, 59].

### Fungal species and skin cancer

The characterization of gut mycobiome distribution in melanoma patients revealed richness in α-diversity accompanied by the enrichment of fungal species belonging to Saccharomycetales. Though a principal coordinate analysis [PCoA] of the gut mycobiome showed no significant difference between melanoma patients and healthy individuals, combined fungal and bacterial diversity showed significant variation [60]. Shiao and colleagues also provided evidence supporting fungal dysbiosis in cancer patients, where the administration of antibiotics in C57BL/6 mice, before the subcutaneous administration of B16 murine melanoma cells, increased the population of *C. albicans* and *Saccharomyces* sp. *C. albicans* and *Saccharomyces* sp. are known to increase the probability of tumor regrowth by enhancing the expression of PD-1^+^ CD8^+^ T cells [61]. Independent research conducted in Taiwan demonstrated a higher risk of non-melanoma skin cancer among patients with *C. albicans* infection compared to those without it [62], which could be because of shared risk factors like immunosuppression [48].

### Fungal species and lung cancer

The risk for lung cancer, a leading cause of death around the globe, has been associated with smoking and inhaling pollutants as well as with somatic and germline mutations [63]. The Mansoura University-approved screening of the pattern of fungal populations from a bronchoalveolar lavage culture of 100 patients with central bronchial carcinoma revealed that *Aspergillus* sp. and *C. albicans* were predominant [64]. In another study, conducted at Central South University, China, a metagenome sequencing-based verification of the presence of key fungal species was done in 66 patients. Higher fungal α and β-diversity were observed in the case of non-small-cell lung cancer (NSCLC) than in non-NSCLCs. Around 45 fungi were found to be associated with the development of NSCLC, with *Alternaria arborescens* being the key fungus [65].

In spite of these findings, a thorough analysis of tumor-associated fungi is still lacking. Further investigation holds promise for the discovery of fungi in diagnostic and prognostic capacities for the above-mentioned cancer types. Recent reports suggest the role of fungi in almost all cancer types. The fungal species associated with different cancer types are summarized in Table 1.

**Table 1:** Alterations in the composition of mycobiome responsible for oncogenic development.

Cancer type	Alternation in mycobiome
Colorectal	Decrease in the richness of *Saccharomyces cerevisiae*, *Lipomyces starkeyi* and *Pneumocystidomycetes* fungi. Abundance of *Malasseziomycete* fungi. Increase in *Basidiomycota*-to-*Ascomycota* ratio
Pancreatic ductal adenocarcinoma	Increase in *Malassezia globose*, *S. cerevisiae*, and *Aspergillus* species.
Gastric	Increases in *Candida* sp. and *Alternaria* sp. Decrease in *Saitozyma* sp. and *Thermomyces* sp.
Oral	Prevalence of *Candida* sp.
Esophageal	Presence of *C. albicans*, *Phialemonium* sp., *Aspergillus* sp., *Penicillium* sp., *Fusarium* sp.
Lung	Prevalence of *Aspergillus* sp., *Cryptococcus* sp., *Pneumocystis* sp. *Trichosporon* sp., *Fusarium* sp., *Rhizopus* sp., *Histoplasma capsulatum*, *H. immitis*, *P. jiroveci*, *T. marneffei*
Breast	*Aspergillus* sp., *Coccidioides* sp., *Geotrichum* sp., *Pleistophora* sp., *Rhodotorula* sp., *Filobasidiella* sp., *Mucor* sp., *Trichophyton* sp., *Epidermophyton* sp., *Candida* sp.*, Pseudallescheria* sp., *Penicillium* sp., *Ajellomyces* sp., *Alternaria* sp., *Piedraia* sp., *Malassezia* sp.*, Cunninghamella* sp.*, Rhizomucor* sp.*, Fonsecaea* sp.
Cervical	*Candida* sp., *Malassezia* sp., *Sporidiobolaeae* sp., *Saccharomyces* sp., *Nakaseomyces* sp., *Gjaerumia* sp., *Pleosporales* sp., *Cryptococcus laurentii*
Ovarian	*Acremonium* sp., *Cladophialophora* sp., *Malassezia* sp., *Microsporidia pleistophora*, *Ajellomyces* sp., *Aspergillus* sp., *Candida* sp., *Cladosporium* sp., *Coccidioides* sp., *Cunninghamella* sp., *Issatchenkia* sp., *Nosema* sp., *Paracoccidioides* sp., *Penicillium* sp., *Pleistophora* sp., *Cryptococcus* sp., *Rhizomucor* sp., *Rhizopus* sp., *Rhodotorula* sp., *Trichophyton* sp.*, Pneumocystis* sp.
Skin	*Cryptococcus neoformans*, *C. immitis*, *Trichosporon* sp., *Mucor* sp., *Rhizopus* sp., *C. albicans*, *C. tropicalis*, *C. glabrata*, *C. parapsilosis, Histoplasma capsulatum, Fusarium* sp.
Gallbladder	*Aspergillus* sp., *A. flavus, A. parasiticus, Penicillium, C. albicans, C. glabrata, C. tropicalis, C. neoformans, Blastomyces dermatitidis*
Prostate	*Aspergillus sp., Candida sp., C. neoformans, H. capsulatum, B. dermatitidis, C. immitis*

Compiled from ref. [31].

## Fungal differentiation and pathogenesis

The ability of fungal species to undergo a reversible phenotypic shift [66] from yeast, consisting of solitary cells [67], to hyphae, characterized by chains of tubular cells [68], is a well-established virulence trait [37]. Y-H morphogenesis in fungi [69–71], where mutants defective in Y-H differentiation display a loss of pathogenicity, reinforcing the crucial role of the hyphal form in fungal virulence [72].

Crucial for dissemination via circulation [73], the adhesion of the yeast form to the endothelium of blood vessels is accomplished by adhesin proteins [74]. In contrast, the hyphal form, mediated by the cAMP-dependent protein kinase-A (PKA) pathway, the mitogen-activated protein kinase [MAPK] cascade [75], and the calcium calmodulin-mediated signal transduction pathway, possesses the ability to invade the protective layers either by active penetration or by induced endocytosis [76]. This establishes the initial stages of infection [24] and resistance towards phagocytosis by volume expansion [77] and by disrupting the structural integrity of immune cells [78]. The crosstalk between the above signaling pathways regulates transcription factors responsible for controlling the expression of hyphal-specific genes such as *als3* [adhesin] [79], h*wp1* [invasin] [80], and h*gc1* [hypha-specific cyclin] [81]. Further, a cause–effect relationship has been reported between genes coding for nitrogen metabolizing enzymes such as glutamate dehydrogenases (GDH EC 1.4.1.3), ornithine decarboxylases (ODC EC 4.1.1.17), and chitin biosynthetic enzymes such as chitin synthases (CHS EC 2.4.1.16).

## Fungal differentiation and cancer development and progression: possible linkages

**Fig. 1:**
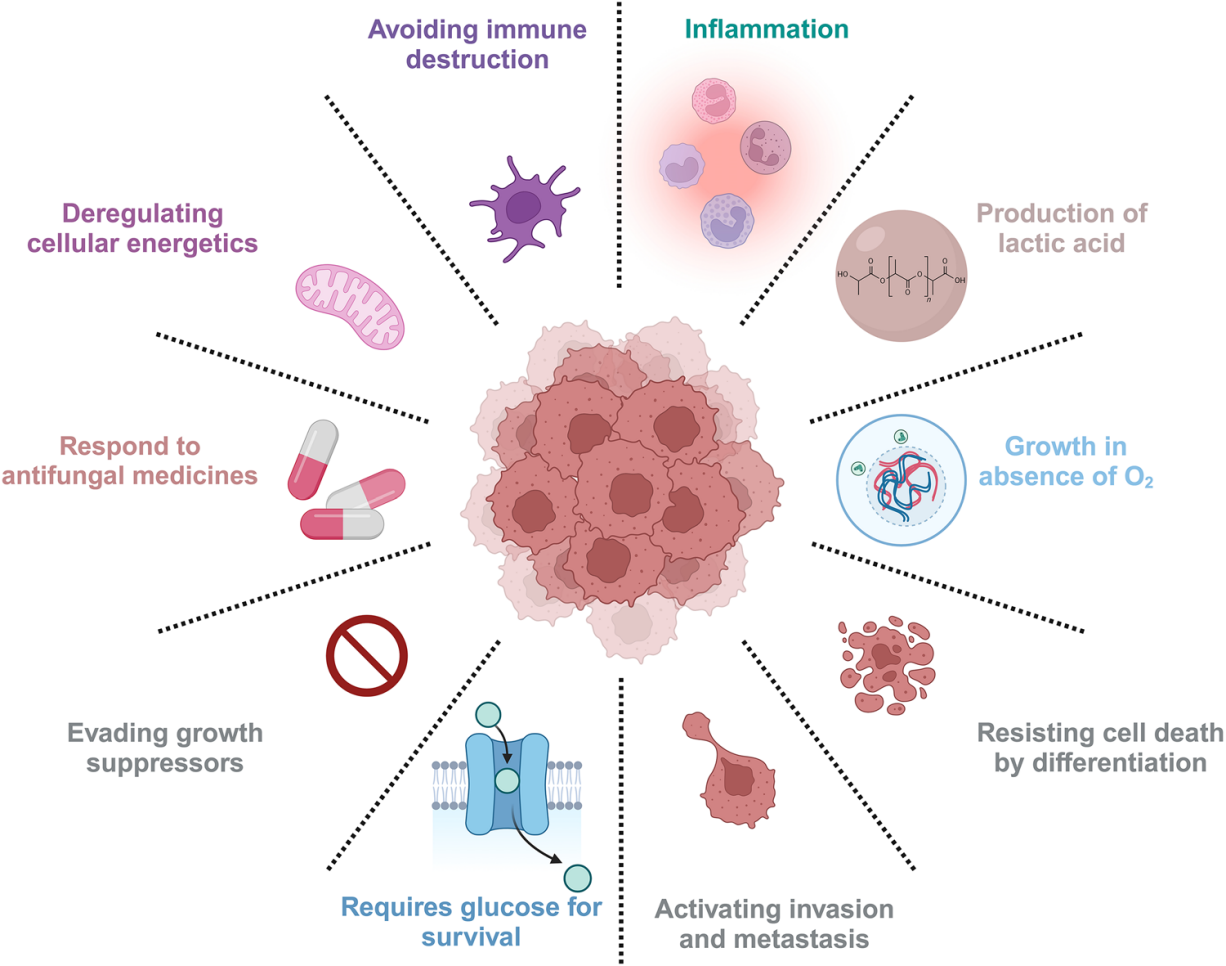
Commonality between differentiating fungal and cancer cells. Overlapping physiochemical properties essential for fungal Y-H transition, cancer cell formation, and progression.

In recent studies, multiple physicochemical properties of cancer and differentiating fungal cells have been found to overlap (Fig. 1). The characteristic features associated with the tumor microenvironment, like excessive production of ROS [82], elevated carbon dioxide levels [18, 83] and increased response to glucose [82] and *N*-acetylglucosamine [54, 84], are also responsible for apical extension during hyphae formation in fungi.

**Fig. 2:**
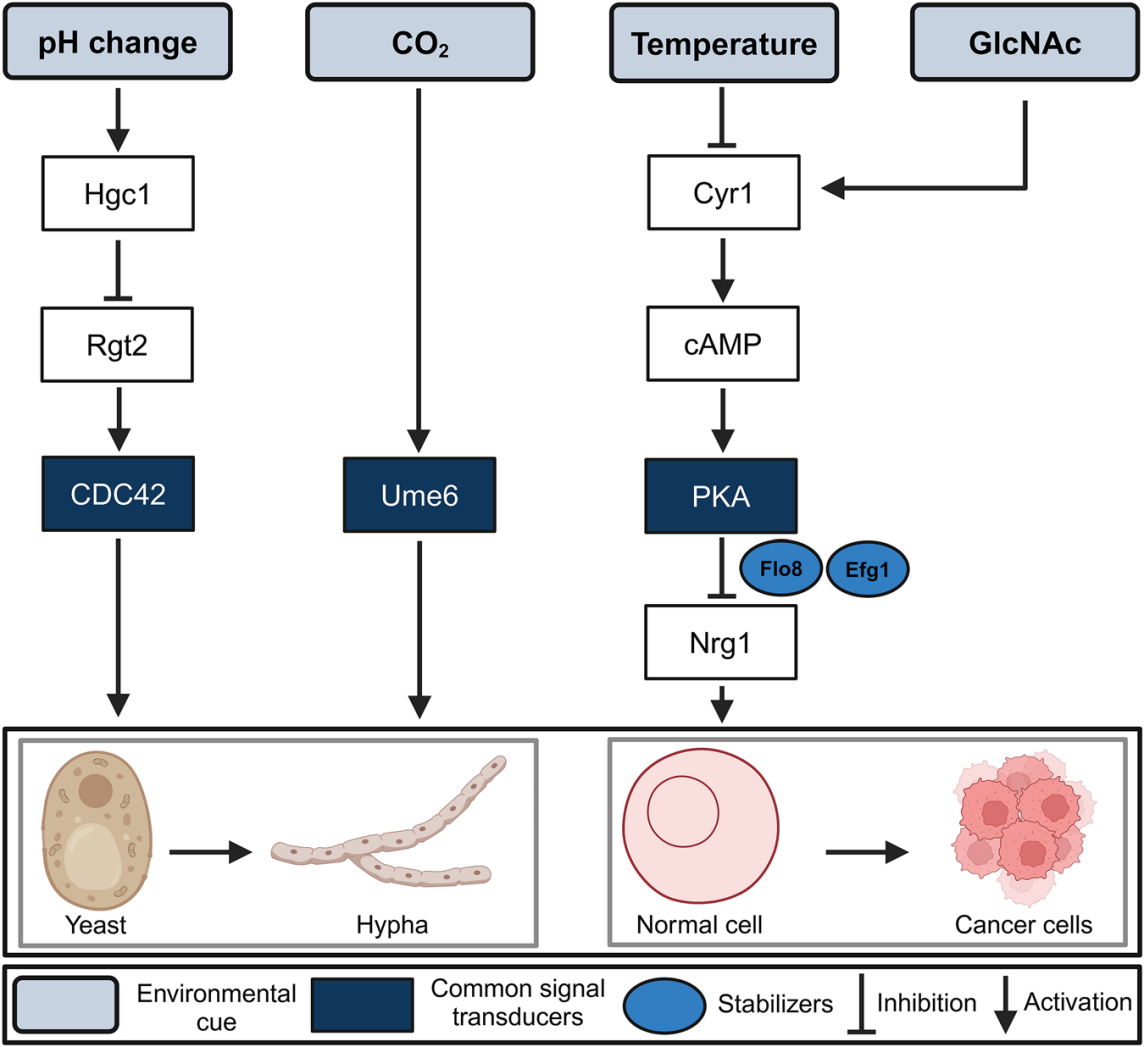
Signal transduction pathways in fungal differentiation and tumor formation. Signal transduction pathways depicting common signals responsible for fungal differentiation and tumor formation.

Other than physicochemical similarities, some signaling pathways responsible for regulating fungal differentiation also overlap with pathways involved in the formation of tumor cells and their progression (Fig. 2) [21, 22]. For example, a component of the cAMP/PKA pathway, *Cyr1* or *Cdc35*, is an adenylyl cyclase that catalyzes cAMP biosynthesis. In turn, cAMP induces conformational changes in PKA to release its catalytic subunit from the regulatory subunit [85]. PKA activates the downstream transcription factor, *Efg1*, to induce the expression of hyphae-specific genes [81]. The cAMP-mediated PKA pathway has been shown to promote tumor cell proliferation and progression also in multiple preclinical cancer models [18].

Cell cycle regulators like cyclins and CDKs, important in the formation or suppression of tumors, are also involved in fungal differentiation [86, 87]. In fungi, hyphal tip growth is characterized by the action of CDC42 in the apical zone. The sustained activation of CDC42 is achieved by the expression of the G1-type cyclin protein, Hgc1 [CDK1 ^Hgc1^], which phosphorylates and prevents the inhibitory action of the Rgt2 GTPase-activating protein [GAP]. Therefore, Hgc1 maintains CDC42-GTP activity in the apical zone by inhibiting GAP-driven GTP hydrolysis [81]. CDC42 is also known to be involved in cancer development and metastasis by the fasciculation of F-actin in the filamentous pseudopodia of the cells [88]. It has also been observed that the myeloid-derived suppressor cell, a potent immunosuppressor, is associated with the onset of tumors [89].

Further, it is reported that fungal dysbiosis can trigger the multiplication of myeloid-derived suppressor cells to contribute to the development of colorectal cancer by promoting an immunosuppressive action that triggers pyruvate kinase M1/2-dependent glycolysis [16]. This aids in the expression of *ume6*, a filament-specific transcription regulator in fungi. The rise in *ume6* level corresponds to the Y-H fungal differentiation, which is involved in tumor metastasis [90].

Besides genes and proteins, non-coding RNAs *viz*. microRNAs (miRNAs), long-non-coding RNAs (lncRNAs), and circular RNAs (circRNAs) are important in multiple biological processes, and play a crucial role in fungal differentiation as well as cancer development (Table 2). Generally, microRNAs are 20–30 nucleotides in length. They exert either transcriptional repression and deadenylation-mediated gene silencing by binding to the 3′ untranslated region (3′UTR) of the target mRNA or induce transcription via interaction with the promoter region [91]. Emerging evidence suggests that noncoding RNAs can control fungal pathogenesis through the formation of an active RISC to induce post-transcriptional gene silencing in a process called RNA interference [92].

**Table 2:** The role of noncoding RNAs in the development and progression of different cancer types.

Non-coding RNAs	Description	Reference
DNA damage-inducible non-coding RNA [DINOR]	Deletion causes DNA damage, which activates RAD53-dependent responses, causing filamentation	[188]
*RZE1*	Deletion causes a reduction of *ZNF2* transcripts, arresting *C. neoformans* in their virulent yeast form	[189]
LincR-PPP2R5C	Deletion causes a significant reduction in *C. neoformans* infection by enhancing the IL-4-mediated fungicidal activity of neutrophils	[190]
LncSSBP1	Deletion enhances the interaction between IL-6 and hnRNPK (heterogeneous nuclear ribonucleoprotein K) to boost the innate immune response against *Talaromyces marneffei*	[191]
*LINC00511*	Regulates breast cancer cell invasion	[192]
*BVES-AS1*	Promotes tumor cell viability, migration, and invasion in vitro	[193]
*KDM4A-AS1*	Weakens cancer cell viability and migration capacity	[194]
MiR-124	Higher expression of miR-124 inhibits spermidine oxidase [SMOX] mediated DNA damage in *H. pylori*-associated gastric cancer	[195]
LINC00265	Regulates microRNAs-based proliferation, migration, invasion, and angiogenesis in osteosarcoma.	[196]
miR-3613-3p	In-vivo overexpression inhibits triple-negative breast cancer [TNBC] tumorigenesis. In vitro overexpression enhances the sensitivity of TNBC to Palbociclib treatment	[197]

Besides in fungal pathogenesis, miRNA expression was dysregulated in cancer cells also. In humans, around 50% of miRNA was found to be damaged or amplified in multiple cancer types [93]. Among these oncogenic miRNAs, the ectopic expression of miR-155 was found to be associated with the development of lymphoblastic leukemia and lymphoma in transgenic mice models [94]. The overexpression of members of mir-17-92 was observed in multiple cancers, including lung and breast cancer [95].

Yet another form of non-coding RNAs is the circRNA. Recent research has shown that circRNAs bind to miRNAs and thus repress and regulate the function of miRNAs. Studies show that circRNAs are involved in several cancers [96]. However, commonalities in specific circRNAs in cancers and in fungi have not been investigated. Investigating this gap in our knowledge may also prove to be fruitful for future research.

lncRNAs have been shown to be involved in the development of several cancers [97, 98]. Specifically, the loss of the nuclear-localized lncRNA, *rze1*, has been shown to reduce the transcript level of the morphogenesis regulator, *znf2*, arresting the cells in their highly virulent yeast form [99]. Additionally, single-molecule fluorescent in-situ hybridization-based microscopic evaluation also indicated that the loss of *rze1* increases the level of *znf2* transcripts in the nucleus compared to the case of the cytoplasm, suggesting the role of lncRNA in controlling the Y-H morphological transition through the well-characterized morphogenesis regulator, *znf2* [100]. Later, the observations of another group regarding the co-expression of lncRNAs with protein-coding transcripts suggested the possibility of the potential functional role of lncRNAs. Here, Hovhannisyan et al. followed a bioinformatic-based approach to understand the expression profile of lncRNAs in different fungal infections of epithelial cells. The analysis showed modules containing pathogenicity-specific lncRNAs with various enrichments. For example, the co-expression of two lncRNAs, MSTRG.6541.1 and MSTRG.6542.1, with an infection-specific protein coding gene CTRG_00938 in *C. tropicalis* further strengthens the hypothesis of co-expression and lncRNAs, with the genes involved in pathogenesis. Apart from fungal pathogenesis, ncRNAs, like miRNAs, contribute to cancer development and progression by interacting with known cancer networks. The oncogenic miRNAs of the miR-17-92 cluster suppress negative regulators of phosphatidylinositol-3-OH kinase signaling or pro-apoptotic members of the BCL-2 family, which disrupts the processes known to influence cancer development. Hence, further strengthening the understanding of the roles of various classes of ncRNAs involved in regulating the common processes in cancer development and fungal pathogenesis could help to design treatment strategies such as ncRNA mimics or antagonist [101]. Additionally, these oncogenic ncRNAs can also be blocked using locked nucleic acid constructs and antisense nucleotides [102].

Hence, lncRNAs can lead to fungal dysbiosis by influencing morphological transitions, which may also initiate cancer cell formation. A comprehensive search for common sequences among fungal lncRNAs and lncRNAs in cancer tissues may be needed to fill in the gaps in research in this direction.

The involvement of lncRNA in cancer has so far been a correlation. The mechanism of action has not yet been revealed completely. Recent reports suggest that at least some lncRNAs have open reading frames and that they may actually code for mini peptides that are bioactive. Already there are indications that some mini peptides are involved in cancers and that they play a role in fungi too. However, due to limited understanding of the amino acid sequences encoded by lncRNA and their involvement in fungal differentiation or in cancer cell progression, the causal factors behind the correlations of lncRNA with these processes is yet to be established. In short, more studies are required to map the roles of miRNA, siRNA, lncRNA, and circRNA in regulating the common processes in cancers and in fungal pathogenesis.

## Fungal differentiation, tumor formation, and apoptosis

**Fig. 3:**
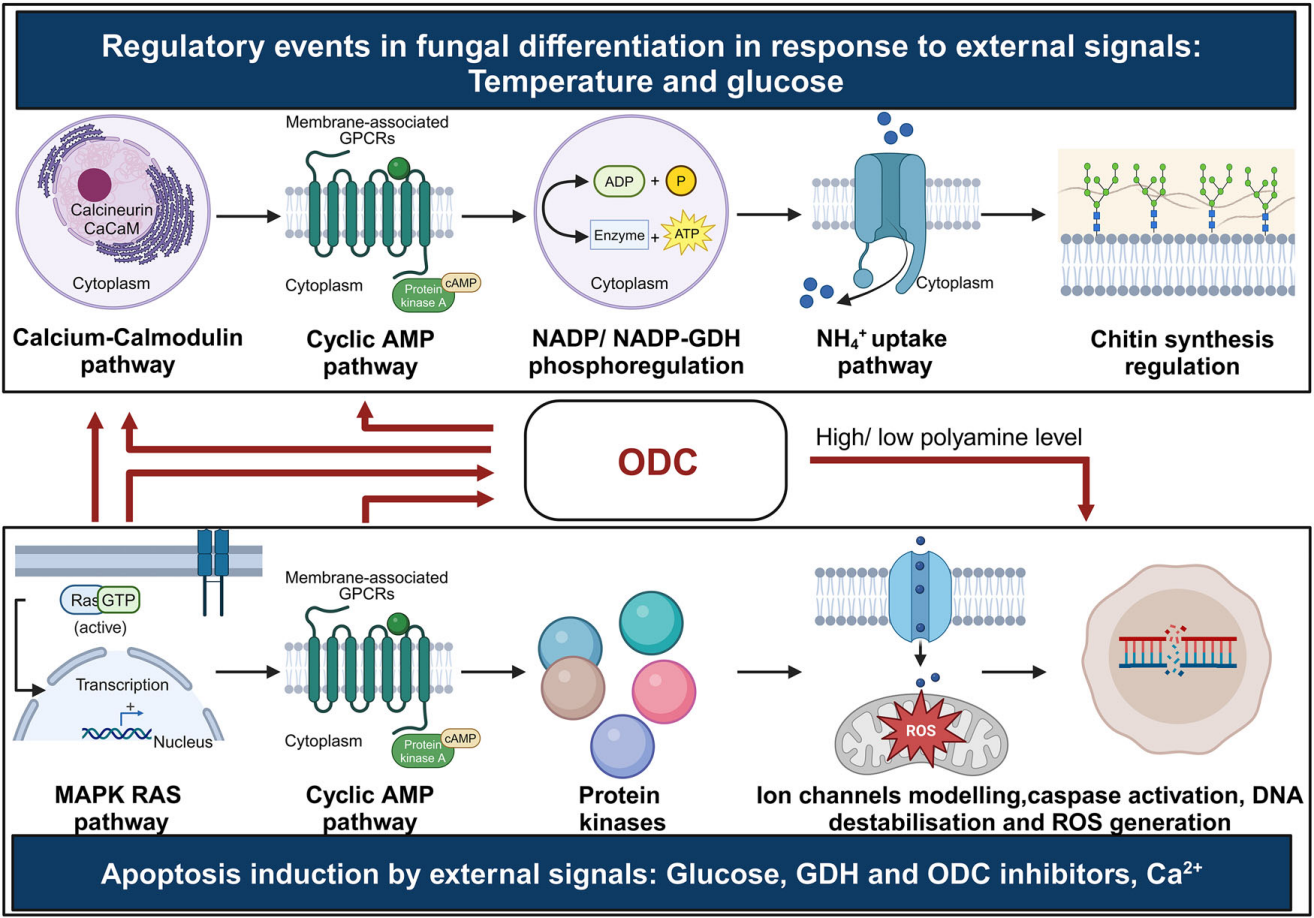
Possible linkage of fungal differentiation with apoptosis. Diagram depicting common molecular events regulating differentiation and apoptosis in fungi, indicating the possible linkage between the two processes.

Most human pathogenic fungi are dimorphic and switch between Y-H growth forms for survival and proliferation in the host, a most important virulence trait [37]. The biochemical and molecular events that regulate differentiation and apoptosis (programmed cell death) in fungi show an overlap, indicating the possible linkage between these two processes (Fig. 3) [21, 22]. The role of apoptotic processes in fungal growth, virulence, competitiveness, and ageing have been documented [103]. Studies suggest that inhibiting differentiation in fungi induces apoptosis [104]. On the other hand, defects in apoptosis induce tumor cell formation in multicellular organisms [105]. If that is so, understanding the process of fungal differentiation, per se, could be useful in designing apoptosis inducers for the development of antifungal as well as anticancer therapies. For instance, the level of polyamines, fungal differentiation, formation of tumor cells, and apoptosis are closely linked [106–109]. The enzyme of polyamine biosynthesis metabolism, ornithine decarboxylase (ODC, EC 4.1.1.17), showed a cause-effect relationship with Y-H morphogenesis in fungi [110]. Knockout mutants of *odc* were arrested in the Y-form and were unable to undergo the Y-H transition [110]. The level of polyamines was found to be higher in hyphae than in yeast cells [111]. In tumor cells also, the expression level of ODC and the concentration of polyamines were found to be elevated [112]. The increased level of polyamines causes DNA fragmentation, chromatin condensation, and the generation of ROS, leading to apoptotic death [113, 114].

In ageing yeast, spermidine treatment triggered the deacetylation of the histone, H3, through the inhibition of histone acetyltransferases (HAT EC 2.3.1.48), suppressing oxidative stress and inducing apoptosis [115]. These observations also suggest that molecules inhibiting the process of differentiation can induce apoptosis in fungi and can thus serve as novel antifungal agents. For instance, molecules like acetic acid, viscosinamide, amphotericin B, and farnesol that exhibit antifungal activity were reported to induce apoptosis in *C. albicans*, *Rhizoctonia solani*, *S. cerevisiae*, *Zygosaccharomyces bailii*, and others [116–118]. These molecules exert their apoptotic effect via caspases, superoxide dismutases (SOD EC 1.15.1.1), and ODCs, which are known biochemical correlates of fungal differentiation [119].

Besides biochemical and molecular processes, the signaling pathways leading to fungal differentiation, tumor cell formation, and apoptosis also overlap. For instance, in the human pathogens, *C. albicans*, *S. cerevisiae*, and the model dimorphic fungus, *Benjaminiella poitrasii*, PKA, Ras, and MAPK signaling networks were shared with the differentiation and apoptotic pathways [21, 22]. Ras knockout mutants of *C. albicans* and *S. cerevisiae* failed to undergo apoptosis even in the presence of apoptotic stimuli. Chou et al. showed that the downregulation of protein kinase C (PKC) levels can induce apoptosis via caspase activation in fungal cells. PKCs belong to a family of phospholipid-dependent serine/threonine kinases that have important roles in signal transduction pathways (Ras and MAPK) and are involved in growth, differentiation, and cell death [120]. In *S. cerevisiae*, the MAPK cascade regulates cell wall biosynthesis, a key determinant of cell morphology, as well as the overall cell cycle. In *C. albicans*, MAPK showed a correlation with hyphae formation [121]. Further, Ras was involved in filamentation, hyphal growth, and mating in fungi such as *Paracoccidioides brasiliensis*. However, its explicit role in the Y-H transition is yet to be explored [121]. Furthermore, the cAMP-dependent PKA cascade regulates growth and virulence in *P. brasiliensis*, where the addition of cAMP inhibits the Y-H transition in this fungus. In the dimorphic fungus, *B. poitrasii*, cAMP and calcium-calmodulin-calcineurin-dependent pathways seem to be involved in differentiation [121].

Several signal transduction pathways involved in fungal differentiation also play key roles in cancer progression, highlighting the commonality between them. For instance, in the last two decades, a number of studies suggest that PKCs, an integral part of Ras and MAPK signaling, are crucial factors in carcinogenesis and, depending on their levels in the cell, they either promote or suppress tumor cell growth and metastasis [122]. The MAPK pathway, crucial for morphogenesis and stress responses in fungi, also regulates cancer cell proliferation and metastasis. The cAMP/ PKA, which controls fungal dimorphism, similarly promotes cell survival and immune evasion in cancer. The PI3K/Akt/TOR pathway, essential for fungal growth, drives tumor progression and drug resistance [123]. Additionally, polyamine biosynthesis via ODC supports fungal proliferation, while in cancer, it fuels cell growth and metastasis [124]. The TGF-β pathway, involved in fungal biofilm formation also drives epithelial-to-mesenchymal transition and immune suppression in cancer [125].

Polyamines, a class of aliphatic metabolites, are essential for the growth and proliferation of both prokaryotic and eukaryotic organisms. Polyamines like spermine and putrescine are involved in multiple biological processes, and their biosynthetic pathway in fungi is quite similar to that in animals. An initiator and rate-limiting enzyme, ODC is a pyridoxal-5′-phosphate-dependent enzyme. It catalyzes the decarboxylation of ornithine to an intermediate diamine putrescine. The spermidine synthase (EC 2.5.1.16) mediated addition of aminopropyl from decarboxylated s-adenosylmethionine led to the synthesis of triamine spermidine and, finally, spermine synthase (EC 2.5.1.22) transfers an additional aminopropyl group to form the tetramine spermine [126]. Several studies have demonstrated the crucial role of polyamines in multiple differentiation events. An experiment executed with *odc* minus mutants of filamentous fungi, *Ustilago maydis* and *Yarrowia lipolytica*, displayed the inability to execute morphogenetic switches in the limiting concentrations of putrescine. However, the mechanism is still unclear [127].

Crucial for multiple biological mechanisms like replication, transcription, translation, and post-translational modifications, polyamines have been frequently reported to promote tumorigenesis [128]. In the hypoxic nature of the tumor microenvironment, the ability to synthesize polyamines decreases, whereas its environmental uptake increases. This disturbance in polyamine homeostasis leads to the decreased production of tumoricidal cytokines and adhesion molecules, leading to the migration of cancer cells [129]. These observations further strengthen the idea of fungi-cancer linkage and suggest that dealing with fungi can have bearing on cancer therapy.

As multiple signaling molecules are common in fungal differentiation and tumor formation, the use of fungal morphogenesis inhibitors could be an effective strategy to downregulate signal transducers involved in tumor development. By disrupting these pathways, it may be possible to alter the tumor microenvironment, reducing conditions favorable for cancer progression and metastasis and ultimately promoting apoptosis in cancer cells [130]. Further, a combination of molecules that target fungal differentiation and trigger tumor cell apoptosis could be a highly effective way to treat cancer.

## Using existing antifungal drugs in anticancer therapy: a repurposing approach

The fungal cell wall, mainly composed of glucans, glycoproteins, chitin, and melanin [131], is a complex structure that plays a crucial role in fungal cell integrity. It is essential for controlling vesicular transportation [132] and for protection against osmotic and mechanical stress [133]. The immune system easily recognizes one of its key components, the polysaccharide, β-glucan. This recognition triggers an immune response from the host against the fungal invaders. To reduce the probability of recognition by the host’s defense system, fungal species follow the strategy of masking the protective sheath [134]. Thus, mannoproteins containing O-glycosylated oligosaccharide and *N*-glycosylated polysaccharide moieties [135] build a mask around the β-glucan layer to suppress recognition and the dectin-1 mediated immune response against fungi [136].

Similarly, chitin, derived from *N*-acetylglucosamine [137], blocks the recognition of *C. albicans* by peripheral blood mononuclear cells and macrophages and, hence, affects cytokine production [138]. It also triggers an alteration in arginase-1-induced nitric oxide production in host macrophages to hamper macrophage-mediated antimicrobial activities [139].

The other component of the fungal cell wall, melanin, is polymerized from phenolic or indolic compounds. Melanin is a negatively charged, hydrophobic pigment essential for survival in the host system [140]. It contributes to fungal virulence [141] and dissemination [142], as well as the inhibition of phagocytosis [131]. Melanized conidia, engulfed by host macrophages, has been found to inhibit their apoptosis by preventing the activation of the executioner caspase [143].

**Fig. 4:**
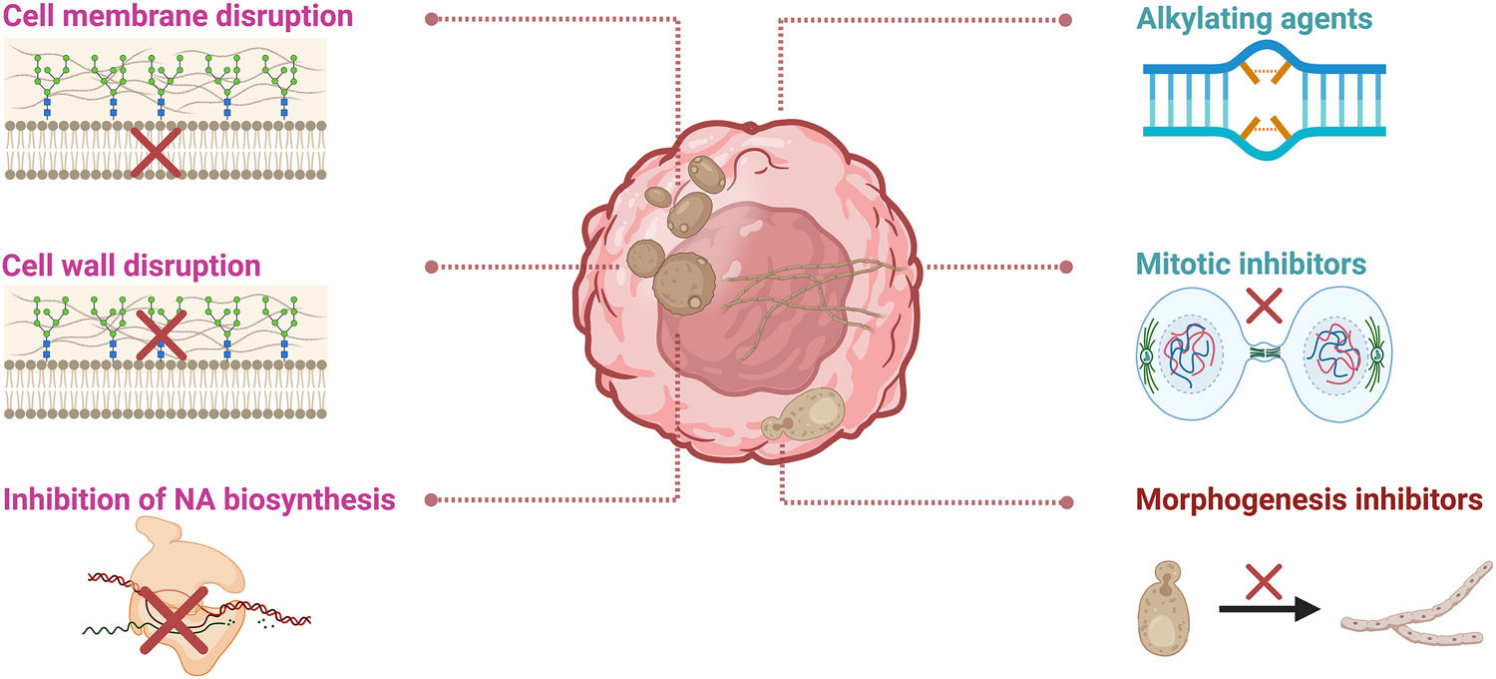
Mechanisms of action of existing antifungal drugs. Cell membrane: Azoles inhibit the synthesis of ergosterol (major sterol), allylamines cause ergosterol depletion by blocking the synthesis of its initial precursor, lanosterol and polyenes, lead to the formation of pores in the membrane, which results in the leakage of ions and ultimately causes cell death. Cell wall: Chitin biosynthesis inhibitors such as nikkomycin block the active site of chitin synthase, altering the activity essential to maintaining the structural integrity of the cell wall. Morphogenesis: Inhibitor such as trifluoperazine inhibit calmodulin-dependent Ca^2+^-ATPase and endocytic pathways essential for hyphal morphogenesis responsible for fungal pathogenesis.

Ergosterol, a 5,7-diene oxysterol, an essential constituent of the fungal cell membrane, controls membrane fluidity and structural integrity [144]. It has been used to induce pyroptosis or caspase-induced inflammatory programmed cell death [145, 146]. Research also suggests that fungal ergosterol may trigger macrophage pyroptosis. The mechanism of existing antifungal drugs is summarized in Fig. 4.

**Fig. 5:**
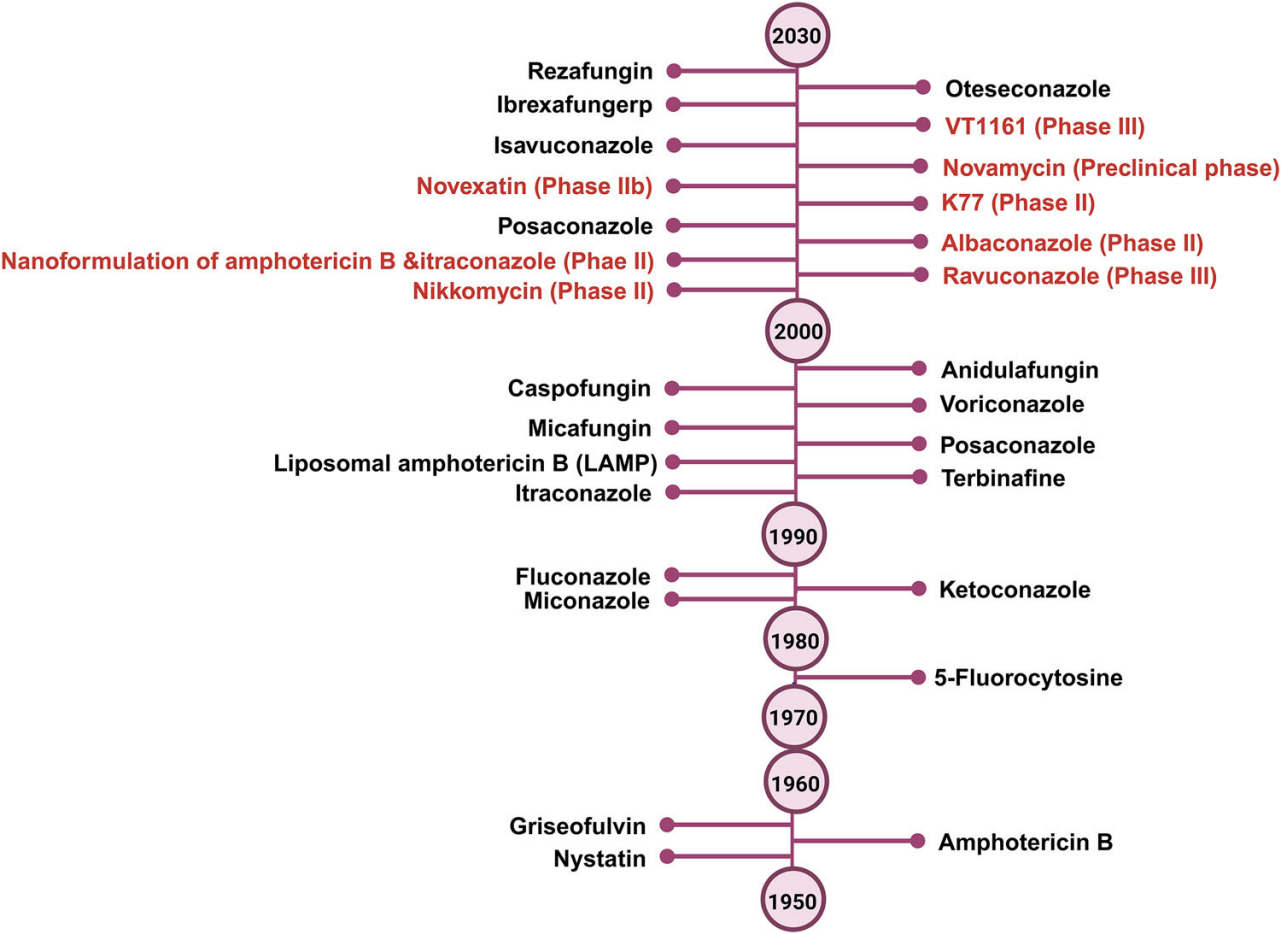
The antifungal drug development pipeline. Antifungal drugs approved by the FDA and under clinical trials (denoted in red font).

Most clinically approved antifungal drugs target either fungal cell walls (chitin, glucan and mannan) or cell membrane (ergosterol)-associated entities, as they are essential for fungal survival and growth, but not necessary for the host [131, 144]. Most antifungals in the pipeline also target either the fungal cell wall or the cell membrane (Fig. 5). Antifungal agents like echinocandins and triterpenoids target fungal cell walls by inhibiting β-D-glucan synthase. Besides, inhibitors targeting chitin synthase and glycosylphosphatidylinositol pathways are also being developed [147]. Another crucial component of fungi is the cell membrane. Enriched with diverse lipids, it protects the cell from desiccation. Compounds like serine hydroxymate and hydroxylamine are known to damage the cell membrane by targeting the phospholipid synthase [148]. A derivative of fluconazole, ravuconazole is a triazole compound that shares the azole-specific mechanism of fungal destruction. It induces ergosterol depletion and accumulation of toxic intermediates by blocking an essential ergosterol biosynthesis pathway [149]. On the other hand, pradimicin is a unique class of fungicidal agents derived from broth filtrates of Actinomycetes. It possesses the ability to recognize and bind to the fungal cell wall-associated D-mannosidase and form a ternary complex consisting of pradimicin, D-mannose, and calcium [150]. It disrupts the structural integrity of cell membranes, but due to the incidence of hepatotoxicity, the drug was eliminated from clinical trials [151]. The challenges and limitations associated with currently used antifungal agents are summarized in Supplementary Table S2.

In the following subsections, we will provide a brief account of antifungals to explore the possibility of their usefulness in cancer treatment.

### Antifungals targeting cell wall

#### Inhibitors of β-1,3-glucan biosynthesis

A membrane-integrated enzyme, UDP-glucose (1,3)-D-glucan-β-3-D-glucosyltransferase, referred to as β-1,3-glucan synthase (EC 2.1.4.34) [152], comprises a membrane-associated catalytic subunit, Fks, and a cytosolic regulatory subunit, Rho1 [153]. In its de-ribosylated form, Rho1 actively promotes the synthesis of β-1,3-glucan. The ribosylation of Rho1 serves as an inhibitory mechanism, downregulating the Fks activity [154] of transferring glucose from the donor to the growing glucan chain via β-1,3-glycosidic linkages [155]. Once synthesized and branched with β-1,6-glucan side chains, β-1,3-glucan is connected to other cell wall constituents to provide elasticity and tensile strength [156].

**Fig. 6:**
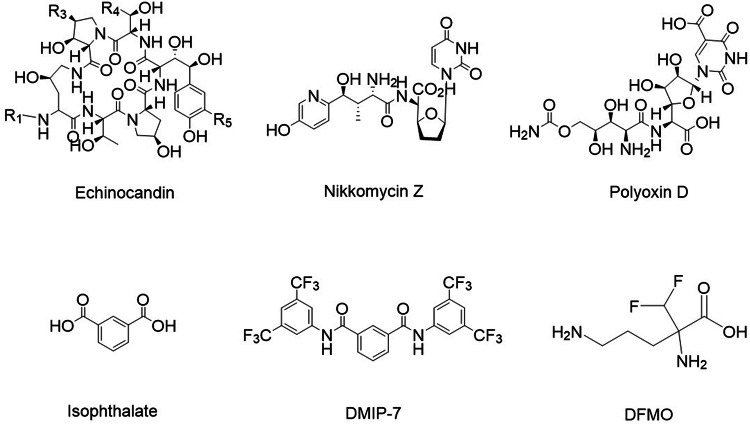
Structure of antifungal agents that target biochemical and molecular correlates of fungal morphogenesis. Echinocandin: Glucan synthase inhibitor; nikkomycin Z and polyoxin D: Chitin synthase inhibitor; isophthalate and dimethyl isophthalate (DMIP): Glutamate dehydrogenase inhibitors; and eflornithine (DFMO): Ornithine decarboxylase inhibitor.

The classes of antifungal compounds responsible for inhibiting β-1,3-glucan synthesis and assembly are acidic terpenoids [enfumafungins], glycolipids [papulacandins] and cyclic lipopeptides [echinocandins]. Echinocandins, derived from *Aspergillus nidulans* [147], are semisynthetic amphiphilic lipoproteins composed of a cyclic hexapeptide core linked to *N*-acyl lipid side chains (Fig. 6) intercalated with the phospholipid bilayer [157] of the membrane. Echinocandins trigger non-competitive inhibition of the catalytic subunit of glucan synthase, resulting in a reduction of β-D-glucan in the cell wall, which leads to structural abnormalities, growth inhibition, and death due to osmotic imbalance [158]. Echinocandins display potent fungicidal activity against most *Candida* sp. and fungistatic action against a few of the *Aspergillus* sp. [159]. However, echinocandins also exhibit hemolytic effects [156]. Hence, cilofungin, an echinocandin B derivative with a 4-octyloxy benzoate side chain, was synthesized but was withdrawn from further studies due to poor water solubility and the toxicity of its co-solvent system containing 26% polyethylene glycol [160].

#### Inhibitors of chitin biosynthesis

Chitin is a homopolymer composed of an *N*-acetylglucosamine monomer conjugated by β-1,4-glycosidic linkages. Composed of multiple antiparallel chains of polysaccharides, chitin is a strong fibrous structure synthesized by multiple sets of enzymes by the utilization of uridine-diphosphate-*N*-acetylglucosamine [UDP-GlcNAc] [137, 153, 161]. The process of chitin biosynthesis is divided into three sub-reactions in which a rate-limiting enzyme, chitin synthase, catalyzes the final reaction, depicting its crucial role in cell development [162]. Peptidyl nucleoside antibiotics like polyoxins and nikkomycins are competitive inhibitors that block this enzyme’s active site, altering the associated activity essential to maintaining the structural integrity of the cell wall (Fig. 6). However, their efficacy as antifungal agents is compromised by their low bioavailability [156].

### Antifungals targeting cell membrane

#### Azoles

The antimycotic agent, azole, functions by targeting the enzyme, cytochrome P-450-dependent 14α-demethylase (EC 1.14.13.70). This enzyme is crucial to the conversion of lanosterol to ergosterol, the predominant sterol in fungal cell membranes [163]. The heterocyclic ring structure of azole containing nitrogen mediates the interaction between the inhibitor and heme moieties of the cytochrome P-450 component of the 14α-demethylase enzyme. The relative affinity of this binding is the determining factor for the efficiency of ergosterol inhibition [164]. Such inhibition leads to the accumulation of sterol precursors and the depletion of an essential component of the fungal membrane [165]. These cellular alterations hamper the structural integrity of the plasma membrane, fungal growth and proliferation, and trigger loss of mitochondrial DNA [166]. Terconazole, efinaconazole, voriconazole, fluconazole, itraconazole, posaconazole, and isavuconazonium are examples of azoles.

#### Polyene

Polyenes are antimycotic agents with a cyclic amphiphilic macrolide substructure derived from the Gram-positive bacteria, *Streptomyces nodosus* [167]. Polyenes, such as natamycin, nystatin B, and amphotericin B are known to disrupt the predominant sterol of the fungal membrane, leading to membrane depolarization and increased Na^+^ and K^+^ transportation and cell death [168]. The hydrophobic interaction between the ring structure, composed of eight amphotericin B molecules and membrane-associated ergosterol, results in the formation of pores with polyene hydroxyl residues facing inward [169]. This arrangement leads to altered permeability, leakage of vital cytoplasmic content, and cell death [170].

#### Allylamine

Allylamines, such as naftifine, butenafine, and terbinafine, inhibit a flavine adenine dinucleotide dependent enzyme, squalene epoxidase (EC 1.14.99.7) [171], responsible for converting squalene to squalene-2,3-epoxide, an intermediate in the biosynthesis of lanosterol [163, 172]. The resulting accumulation of squalene within the fungal cell increases membrane permeability and distorted intracellular organization [168]. Reduced efficacy, systemic toxicity, and acquired resistance, observed in the allylamine class of antifungal drugs, highlight the importance of more potent and efficacious antifungal treatments [173].

### Y-H differentiation as an antifungal target

Besides targeting the cell membrane and associated components of fungi, given the importance of the Y-H phenotypic switch in fungal virulence, targeting this transition can be a promising antifungal therapy. In this pursuit, Bar-Yosef et al. screened around 4000 compounds for their anti-hyphal capacity across a concentration range of 150 to 0.06 μM, revealing 23 compounds (Table 3) that can target morphogenesis at 50 μM or below. Further exploration of the effects of sub-inhibitory concentrations of selected inhibitors, 30 μM trifluoperazine and 20 μM CGS 12066B, on engineered strains carrying maltose-inducible copies of one of three genes [cyclin *hgc1*, transcription factor *ume6*, MAPKK *ste11*], responsible for hyphal formation, revealed complete inhibition of morphogenesis induced by Ste11 and CaUme6 and partial inhibition induced by Hgc. The mechanistic study demonstrated the capacity of these drugs to inhibit calmodulin-dependent Ca^2+^-ATPase and endocytic pathways essential for hyphal morphogenesis. However, the concentration of these inhibitors required to trigger even partial hyphal inhibition was found to be above the established safety threshold of these drugs, which calls for further exploration to find anti-hyphal compounds with lower systemic toxicity [173].

**Table 3:** Effect of different inhibitors on the Y-H transition.

Inhibitor	Concentration [μM]					
	50	25	12.5	6.2	3.1	1.6
5-Nonyloxytrypta-mine	++/R	++/R	+/-/R	-	-	-
Aripiprazole	++/R	+/R	+/-/R	-	-	-
Benproperine	++/R	+/R	-	-	-	-
Bifonazole	+/R	+/-/R	+/-/R	-/R	-	-
Cerivastatin Na	+/R	-	-	-	-	-
CGS 12066B	++/R	++/R	+/	+/-/R	-	-
Clofazimine	+/R	+/-/R	-	-	-	-
Diphenylcyclo-propenone	++/R	+/R	+/-/R	-	-	-
Disulfiram	+/R	+/-/R	-	-	-	-
Ebselen	++/R	+/R	-	-	-	-
Fluphenazine	+/R	-	-	-	-	-
Indatraline	++/R	+/R	+/-/R	-	-	-
Lofepramine	++/R	+/R	-	-	-	-
Naftopidil	++/R	+/R	-	-	-	-
Pergolide	++/R	+/R	+/-/R	-	-	-
Prochlorperazine	++/R	+/R	+/-/R	+/-/R	-	-
Pterostilbene	++/R	+/R	-	-	-	-
Stiripentol	++/R	+/R	+/-/R	-	-	-
Tegaserod maleate	++/R	+/-/R	-	-	-	-
Terbinafine	++/R	+/R	-	-	-	-
Ticlopidine	++/R	+/R	-	-	-	-
Toremifene	++/R	+/-/R	-	-	-	-
Trifluoperazine	++/R	+/R	+/-/R	-	-	-

R (reduction) indicates a visual reduction in hyphal growth. ++ indicates colonies of yeast cells. + indicates higher yeast cells compared to hyphae. +/- denotes higher hyphae than yeast, and - shows hyphae only. Compiled from: ref. [173].

A recent investigation of the cytochalasin actin inhibitor, 19,20-epoxycytochalasin Q [ECQ], isolated from *Xylaria* sp. BCC1067, displayed anti-hyphal capacity against *C. albicans*. A 24h treatment with 256 μg/mL of ECQ inhibited over 95% of hyphal formation. Interestingly, combining ECQ with lipid-based biosurfactants showed enhanced activity under a similar set of conditions [174]. However, while repurposing the existing antifungal agents for anticancer therapy, the challenges and limitations associated with them must also be considered (Supplementary Table S2). Moreover, it would be interesting to check the anticancer potential of inhibitors of biochemical and molecular correlates of fungal morphogenesis (Fig. 6).

## Combination therapies for effective and safe cancer treatment

In the light of evidence on the association between fungal differentiation cum pathogenesis, the development of cancer, and options available to combat fungal infections and cancers, three different approaches to trigger tumor cell apoptosis can be suggested:

1. Since there is a correlation between cancer development and fungal pathogenesis, antifungal drugs can be repurposed to downregulate oncogenes, which will eventually lead to tumor cell apoptosis.
2. For enhanced apoptotic effect, Y-H transition inhibitors can be combined with apoptosis-inducing drugs for the downregulation of common signal transducers (NADP-GDH, ODC, etc.).
3. Chitin and glucan exclusive to fungi can be targeted for the production of antibodies against intra-tumor fungi for selective neutralization, with minimum or no side effects.

The utility of these approaches in target-specific and safe anticancer therapy is discussed in detail in the following sections.

### Repurposing antifungal drugs and their combination with anticancer agents to downregulate oncogenes

The previously mentioned allylamine, responsible for inhibiting fungal growth by targeting the enzyme, squalene epoxidase, is also involved in human cholesterol biosynthesis. In recent years, the relationship between high cholesterol levels and cancer development has been demonstrated [175] and also reconfirmed by the therapeutic potential of cholesterol-lowering drugs like statins in cancer patients [176]. Hence, these biochemical similarities between fungi and their human hosts suggest the utility of repurposing antifungal drugs as anticancer agents. Moreover, the ability of squalene epoxidase to participate in cancer development and migration can be limited by combining allylamine and its derivatives with anticancer drugs to limit the metastasis of cancer cells [177, 178].

Along with its crucial role in ergosterol biosynthesis, cytochrome P450 monooxygenase has also been found to participate in cancer initiation by mediating the conversion of procarcinogens to carcinogens [179]. Cytochrome P450 monooxygenase also alters therapeutic responses by inactivating antitumor agents, leading to the development of chemotherapy resistance [180]. Hence, administering azole antifungals like ketoconazole might help to increase the efficiency of chemotherapeutic agents and antitumor drugs by limiting their catalysis into inactive derivatives [177].

### Combination of existing anticancer drugs and Y-H morphogenesis inhibitors

A recent study on the anti-morphogenetic activity of 2-aryloxazoline derivatives (4i and 9i) showed a dose-dependent reduction in the hyphae formation of both *C. albicans* and *C. tropicalis* [181]. The inhibitory action of 4i was associated with the obstruction of cAMP and MAPK pathways [182] due to the downregulation of the *CEK1* gene involved in hyphal development and virulence. Along with the downregulation of transcription regulators of filamentation (*TEC1*, *EFG1*), other morphogenesis-associated virulence genes (*HWP1*, *ALS3*, ECE1) were also found to be affected [181].

**Fig. 7:**
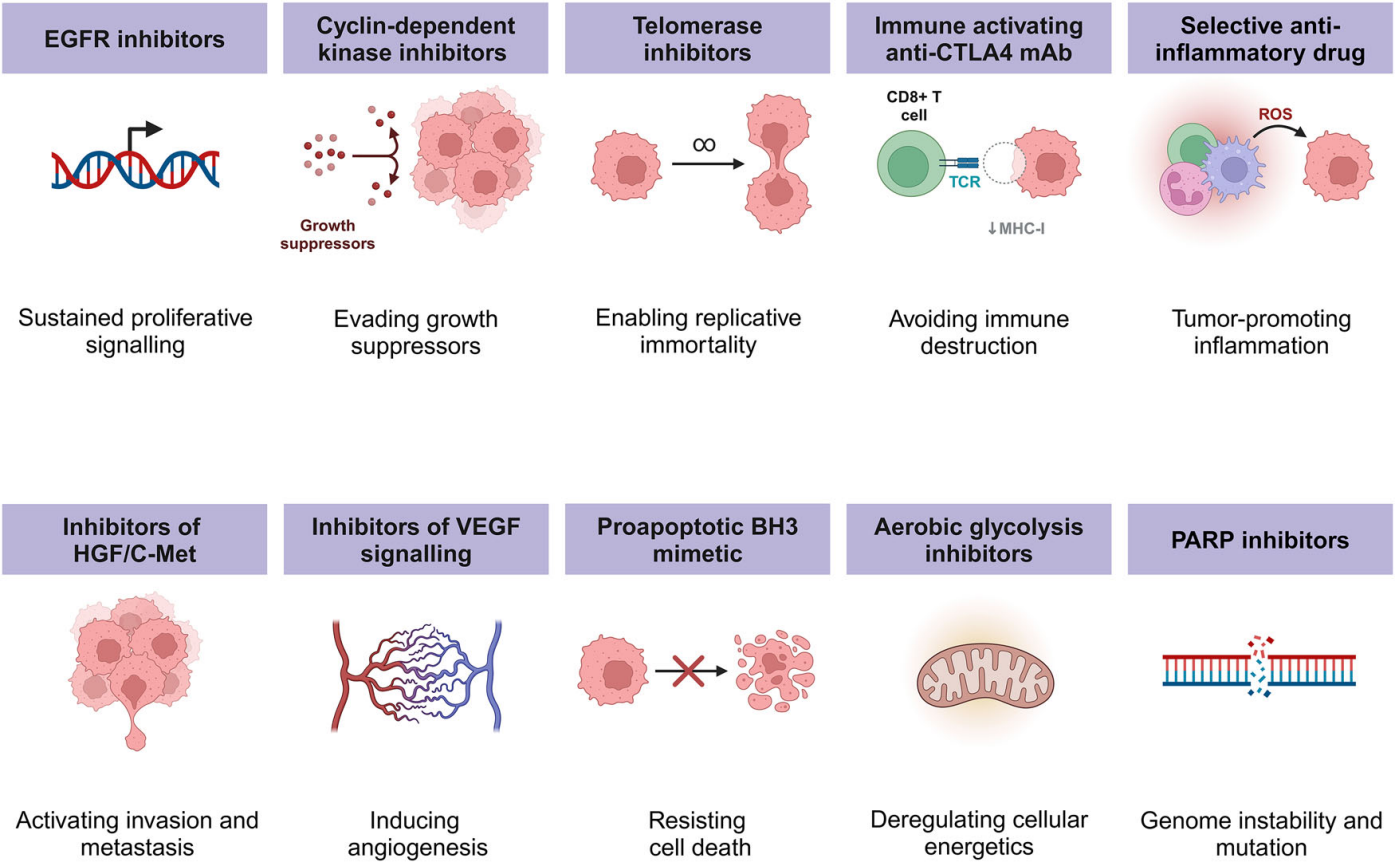
Therapeutic targeting of underlying causes of cancer. Different mechanisms of inducing cancer cell apoptosis through associated hallmark traits (CTLA4 cytotoxic T lymphocyte-associated protein 4, PARP poly (ADP-ribose) polymerase, BH3 Bcl-2 homology, HGF/c-Met hepatocyte growth factor with its receptor c-Met, VEGF vascular endothelial growth factor, EGFR epidermal growth factor receptor).

The therapeutic targets of currently used anticancer drugs are summarized in Fig. 7. For instance, Bruton’s tyrosine kinase (BTK), an essential component of the oncogenic signaling pathway, is crucial in the proliferation and survival of leukemic cells [130]. Targeting BTK with FDA-approved anticancer drugs, such as acalabrutinib and ibrutinib, which are metabolized by the CYP3A enzyme, common to both fungi and humans, can be combined with fungal morphogenesis inhibitors to enhance the apoptotic effect [183].

### Targeting fungal-derived β-glucan in combination with antitumor monoclonal antibodies

**Fig. 8:**
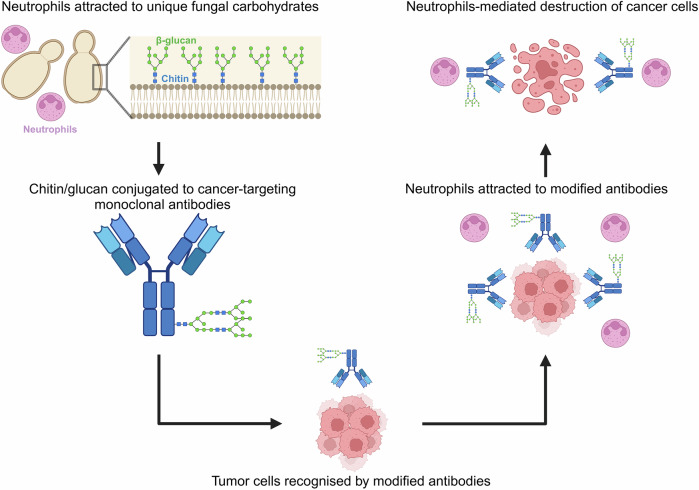
Synthesis of chitin/glucan-associated antibodies to trigger cancer cell apoptosis. The synthesis of monoclonal antibodies conjugated with unique carbohydrates promotes the production and release of carbohydrate-specific neutrophils, which, in turn, trigger cancer cell apoptosis.

Antitumor monoclonal antibodies [mAb] are synthetic protein molecules that selectively target tumor-associated antigens (Table 4). The interaction of mAb with the immune cell-associated Fcγ-receptor [FCGR] facilitates the activation of multiple immune responses, such as the activation of the phagocytic engulfment of antibody-bound tumor cells, a process referred to as antibody-dependent cellular phagocytosis [ADCP]. Additionally, this interaction can stimulate the secretion of innate immune cells to lyse sufficiently opsonized targets, a phenomenon known as antibody-dependent cellular cytotoxicity [ADCC] [184]. These antitumor monoclonal antibodies can be combined with β-glucan synthase inhibitors. The interlinking polymers of the cell wall, β1-3 glucan and β1-6 glucan, are attractive targets for developing antifungal drugs due to their absence in human cells [185], offering the potential for selective neutralization of fungal cells (Fig. 8). β1-3 glucan is synthesized by the membrane-integrated synthase, FKS, by transferring glucose from donor UDP-glucose to the growing chain of glucans *via* β-1,3-glycosidic linkages. Currently, echinocandin and ibrexafungerp are widely used in clinical practice [186, 187] along with the other β-glucan inhibitors mentioned in the section above.

**Table 4:** Tumor-specific FDA-approved monoclonal antibodies.

Antigen category	Antibody	Target antigen	IgG type	Tumor type	Mechanism of action
Hematological cancer	Rituximab	CD20	Chimeric IgG1	Non-Hodgkin lymphoma; chronic lymphocytic leukemia	ADCP, ADCC, CDC
	Ofatumumab	CD20	Human IgG1	Chronic lymphocytic leukemia	ADCP, ADCC, CDC
	Ibritumomab tiuxetan	CD20	Murine IgG1/radioisotope conjugate	Non-Hodgkin lymphoma	Radionucleotide delivery
	Tositumomab-131	CD20	Murine IgG2a/radioisotope conjugate	Non-Hodgkin lymphoma	Radionucleotide delivery
	Obinutuzumab	CD20	Humanized IgG1	Chronic lymphocytic leukemia	ADCC, ADCP
	Alemtuzumab	CD52	Humanized IgG1	B-cell chronic lymphocytic leukemia	ADCP, ADCC, CDC
	Tafasitamab	CD19	Humanized IgG1	Diffuse large B-cell lymphoma	ADCP, ADCC, CDC
	Loncastuximab tesirine	CD19	Humanized IgG1	Diffuse large B-cell lymphoma	Cytotoxic drug delivery
	Polatuzumab vedotin	CD79b	Humanized IgG1 ADC	Diffuse large B-cell lymphoma	Cytotoxic drug delivery
	Daratumumab	CD38	Human IgG1/k	Multiple myeloma	CDC, ADCC, ADCP, neutralization
	Isatuximab	CD38	Chimeric IgG1	Multiple myeloma	ADCP, ADCC, CDC
	Mogamulizumab	CCR4	Humanized IgG1	Cutaneous T-cell lymphoma	ADCP, ADCC, CDC
	Gemtuzumab ozogamicin	CD33	Humanized IgG4/ toxin conjugate	Acute myeloid leukemia [AML]	Cytotoxic drug delivery
	Inotuzumab ozogamicin	CD22	Humanized IgG4 as ADC	B-cell precursor acute lymphoblastic leukemia	Cytotoxic drug delivery
	Moxetumomab pasudotox	CD22	Murine IgG1 dsFv immunotoxin	Hairy cell leukemia	Cytotoxic drug delivery
	Belantamab mafodotin	BCMA	Humanized IgG1 as ADC	Multiple myeloma	Cytotoxic drug delivery
	Brentuximab vedotin	CD30	Chimeric IgG1 as ADC	Hodgkin lymphoma [HL], systemic anaplastic large cell lymphoma [ALCL]	Cytotoxic drug delivery
	Elotuzumab	SLAMF7	Humanized IgG1	Multiple myeloma	ADCP, ADCC, CDC
Solid cancer [ErbB family]	Trastuzumab	HER2	Humanized IgG1	Breast cancer, metastatic gastric or gastroesophageal junction adenocarcinoma	ADCP, CDC^b^
	Ado-Trastuzumab emtansine	HER2	Humanized IgG1 as ADC	Breast cancer	Cytotoxic drug delivery
	[fam]-trastuzumab deruxtecan	HER2	Humanized IgG1 ADC	Breast cancer	Cytotoxic drug delivery
	Pertuzumab	HER2	Humanized IgG1	Breast cancer	Signal blockade, ADCP, CDC^b^
	Margetuximab	HER2	Chimeric IgG1	Breast cancer	ADCP, ADCC
	Cetuximab	EGFR	Chimeric IgG1	Head and neck cancer; colorectal cancer	Signal blockade, ADCC, CDC
	Panitumumab	EGFR	Human IgG2	Metastatic colorectal carcinoma	Signal blockade
	Necitumumab	EGFR	Human IgG1	Carcinoma, non-small cell lung	Signal blockade, ADCC
Solid cancer [other targets]	Dinutuximab	GD2	Chimeric IgG1	Neuroblastoma	ADCC, ADCP, CDC
	Naxitamab	GD2	Humanized IgG1	Neuroblastoma	ADCC, ADCP, CDC
	Enfortumab vedotin	Nectin-4	Human IgG1 ADC	Urothelial cancer	Cytotoxic drug delivery
	Sacituzumab govitecan	TROP-2	Humanized IgG1 ADC	Breast cancer	Cytotoxic drug delivery
			Murine Fab fragment	Colorectal cancer	Detection [nontherapeutic]
	Satumomab	TAG-72	Murine MAb	Colorectal and ovarian cancers	Detection [nontherapeutic]
	Capromab	PSMA	Murine MAb	Prostate adenocarcinoma	Detection [nontherapeutic]

Compiled from: ref. [184]. ^b^Mechanism of action when trastuzumab and pertuzumab are used in combination

### Epilogue

A large number of studies have provided evidence of an increased fungal load, along with an altered mycobiome population, in the tumor microenvironment, suggesting fungal involvement in carcinogenesis. Though numerous studies provide evidence supporting the association between fungal infection and cancer development and progression, much remains to be explored. Future work needs to uncover cancer-type-specific fungal signatures overlapping with tumor cell progression as well as the enzymes, peptides, and various non-coding RNAs that are common to both fungi and cancers. These findings could further substantiate a positive correlation and help identify common molecular targets for combating fungal pathogens along with the induction of tumor cell apoptosis. Besides therapeutic potential, the crosstalk between tumor-associated fungal communities accompanied by bacterial and viral populations can also help in the prognosis and diagnosis of specific cancer types.

## Supplementary information

Supplementary Information
