# Supplementary material for: Intratumor fungi specific mechanisms to influence cell death pathways and trigger tumor cell apoptosis
Source: Cell Death Discov. 2025 Apr 21;11:188. doi: 10.1038/s41420-025-02483-z (PMC12012188; doi:10.1038/s41420-025-02483-z)
Supplement: Supplementary file 1 — Supplementary Information [file 41420_2025_2483_MOESM1_ESM.docx]

**Supplementary Information**

**Intratumor fungi specific mechanisms to influence cell death pathways and trigger tumor cell apoptosis**

Simran S. Ghogare and Ejaj K. Pathan^*^

*Symbiosis School of Biological Sciences, Symbiosis International (Deemed University), Lavale, Pune-412115, Maharashtra, India.*

***Corresponding author**

Symbiosis School of Biological Sciences,

Symbiosis International (Deemed University),

Lavale, Pune-412115, Maharashtra, India

**Email: ek.pathan@ssbs.edu.in**

**Phone: +91-20-28116365**

**Supplementary Table S1.** The molecules and strategies used by fungal pathogens to evade host immune recognition and response

| **Molecules** | **Mechanism to evade immune response** | **Reference** |
| --- | --- | --- |
| Mannans | Mannoproteins containing O-glycosylated oligosaccharide and *N*-glycosylated polysaccharide moieties build a mask around the β-glucan layer to suppress recognition and the dectin-1-mediated immune response against fungi. | (Bain et al., 2014) |
| Chitin | Triggers alteration in arginase-1-induced nitric oxide production in host macrophages to hamper the macrophage-mediated antimicrobial activities. | (Wagener et al., 2017) |
| Melanin | Inhibits NADPH oxidase-dependent activation of phagocytic pathway. | (Chamilos et al., 2016) |
| Titan cells | Impose resistance towards reactive oxygen and nitrogen species (RONS) generated by the host to neutralise pathogens. | (Okagaki & Nielsen, 2012) |
| Asteroid bodies | Interfere with the immune response by trapping IgGs and IgMs. | (Hernández-Chávez et al., 2017) |

**Supplementary Table S2. The challenges and limitations associated with clinically used antifungal agents.**

| **Class** | **Antifungal drug** | **Target fungi** | **Challenges and limitations** | **References** |
| --- | --- | --- | --- | --- |
| Azole | Itraconazole | Filamentous | - Poor absorption of the orally administered formulation. - Probable development of resistance against *Aspergillus* sp. - Interferes with cytochrome p450 enzyme system of potential anticancer drugs such as cisapride, terfenadine, terfenadine, triazolam and midazolam, leading to ventricular arrhythmias and respiratory depression. - Cross-resistance between drugs may lead to increased expression of CgCDR1 and CgCDR2 encoded efflux pumps, reducing efficacy. | (Chiou et al., 2000)    (Roemer & Krysan, 2014)  (Nami et al., 2019) |
|  | Voriconazole | Pathogenic, dimorphic | Display higher MICs against fluconazole-resistant strains, suggesting the possibility of cross-resistance. | (Chiou et al., 2000) |
| Allylamine | Terbinafine | Dermatophytes | Upper respiratory inflammation | (McKeny et al., 2025) |
| Polyene | Nystatin | *Candida* sp. | Lethal and non-effective at maximum tolerated dose [MTD]. | (Chiou et al., 2000) |
|  | Pradimicin | Pathogenic, dimorphic, dematiaceous, zygomycetes | Severe hepatotoxicity | (Chiou et al., 2000) |
| Echinocandin | Caspofungin | *Candida* sp. | Respiratory failure, phlebitis, hypotension | (McKeny et al., 2025) |
|  | Micafungin | *Aspergillus* sp.  *Candida* sp. | Renal failure, anaemia transaminitis, hyperbilirubinemia | (McKeny et al., 2025) |
| Pyrimidine | 5-fluorocytosine | *Cryptococcus* sp. | A resultant toxic compound,  5-fluorouracil interferes with DNA and RNA metabolism. | (Puumala et al., 2024) |

**References**

Bain, J. M., Louw, J., Lewis, L. E., Okai, B., Walls, C. A., Ballou, E. R., Walker, L. A., Reid, D., Munro, C. A., Brown, A. J. P., Brown, G. D., Gow, N. A. R., & Erwig, L. P. (2014). *Candida albicans* hypha formation and mannan masking of β-Glucan inhibit macrophage phagosome maturation. *MBio*, *5*(6). <https://doi.org/10.1128/mBio.01874-14>

Chamilos, G., Akoumianaki, T., Kyrmizi, I., Brakhage, A., Beauvais, A., & Latge, J.-P. (2016). Melanin targets LC3-associated phagocytosis (LAP): A novel pathogenetic mechanism in fungal disease. *Autophagy*, *12*(5), 888–889. <https://doi.org/10.1080/15548627.2016.1157242>

Chiou, C. C., Groll, A. H., & Walsh, T. J. (2000). New drugs and novel targets for treatment of invasive fungal infections in patients with cancer. *The Oncologist*, *5*(2), 120–135. <https://doi.org/10.1634/theoncologist.5-2-120>

Hernández-Chávez, M. J., Pérez-García, L. A., Niño-Vega, G. A., & Mora-Montes, H. M. (2017). Fungal strategies to evade the host immune recognition. *Journal of Fungi*, *3*(4), 51. <https://doi.org/10.3390/jof3040051>

McKeny, P. T., Nessel, T. A., & Zito, P. M. (2025). *Antifungal Antibiotics.*

Nami, S., Aghebati-Maleki, A., Morovati, H., & Aghebati-Maleki, L. (2019). Current antifungal drugs and immunotherapeutic approaches as promising strategies to treatment of fungal diseases. *Biomedecine & Pharmacotherapie*, *110*, 857–868. <https://doi.org/10.1016/j.biopha.2018.12.009>

Okagaki, L. H., & Nielsen, K. (2012). Titan cells confer protection from phagocytosis in *Cryptococcus neoformans* infections. *Eukaryotic Cell*, *11*(6), 820-826. <https://doi.org/10.1128/EC.00121-12>

Puumala, E., Fallah, S., Robbins, N., & Cowen, L. E. (2024). Advancements and challenges in antifungal therapeutic development. *Clinical Microbiology Reviews*, *37*(1), e0014223. <https://doi.org/10.1128/cmr.00142-23>

Roemer, T., & Krysan, D. J. (2014). Antifungal drug development: challenges, unmet clinical needs, and new approaches. *Cold Spring Harbor Perspectives in Medicine*, *4*(5). <https://doi.org/10.1101/cshperspect.a019703>

Wagener, J., Maccallum, D. M., Brown, G. D., & Gow, N. A. R. (2017). *Candida albicans* chitin increases arginase-1 activity in human macrophages, with an impact on macrophage antimicrobial functions. *MBio*, *8*(1). <https://doi.org/10.1128/mBio.01820-16>
